# Sap flow and growth response of Norway spruce under long-term partial rainfall exclusion at low altitude

**DOI:** 10.3389/fpls.2023.1089706

**Published:** 2023-02-14

**Authors:** Ina Zavadilová, Justyna Szatniewska, Peter Petrík, Oldřich Mauer, Radek Pokorný, Marko Stojanović

**Affiliations:** ^1^ Global Change Research Institute, Czech Academy of Sciences, Brno, Czechia; ^2^ Department of Forest Ecology, Faculty of Forestry and Wood Technology, Mendel University, Brno, Czechia; ^3^ Department of Silviculture, Faculty of Forestry and Wood Technology, Mendel University, Brno, Czechia

**Keywords:** tree water use, tree water deficit, stem radial variation, precipitation exclusion, recovery, *Picea abies*, isohydricity, tree ring width

## Abstract

**Introduction:**

Under ongoing climate change, more frequent and severe drought periods accompanied by heat waves are expected in the future. Under these conditions, the tree’s survival is conditioned by fast recovery of functions after drought release. Therefore, in the presented study, we evaluated the effect of long-term water reduction in soil on tree water use and growth dynamics of Norway spruce.

**Methods:**

The experiment was conducted in two young Norway spruce plots located on suboptimal sites at a low altitude of 440 m a.s.l. In the first plot (PE), 25% of precipitation throughfall was excluded since 2007, and the second one represented the control treatment with ambient conditions (PC). Tree sap flow, stem radial increment, and tree water deficit were monitored in two consecutive growing seasons: 2015-2016, with contrasting hydro-climatic conditions.

**Results:**

Trees in both treatments showed relatively isohydric behavior reflected in a strong reduction of sap flow under the exceptional drought of 2015. Nevertheless, trees from PE treatment reduced sap flow faster than PC under decreasing soil water potential, exhibiting faster stomatal response. This led to a significantly lower sap flow of PE, compared to PC in 2015. The maximal sap flow rates were also lower for PE treatment, compared to PC. Both treatments experienced minimal radial growth during the 2015 drought and subsequent recovery of radial growth under the more the humid year of 2016. However, treatments did not differ significantly in stem radial increments within respective years.

**Discussion:**

Precipitation exclusion treatment, therefore, led to water loss adjustment, but did not affect growth response to intense drought and growth recovery in the year after drought.

## 1 Introduction

Norway spruce (*Picea abies* [L.] Karst.) is one of the most important tree species for forestry in Europe (Lu et al., 1996; Spiecker, 2003). Due to its high productivity and economic value (Klimo and Hager, 2000), spruce was widely cultivated outside its natural range and frequently in unsuitable conditions (Spiecker, 2003; Střelcová et al., 2013). It is the most widespread tree species in the Czech Republic, representing almost 50% of forest area and more than 1.3 million hectares, from which only 11% was the natural share of Norway spruce in the species composition (Mansfeld, 2011; Čermák et al., 2021). Due to its high productivity and economic value (Klimo and Hager, 2000), spruce was widely cultivated outside its natural range and frequently in unsuitable conditions (Spiecker, 2003; Střelcová et al., 2013), beyond its ecological and physiological optimum (Modrzyński, 2007). Furthermore, the shift in climatic conditions caused previously favorable sites for Norway spruce cultivation to become unsuitable for this species as its susceptibility to drought stress is commonly reported (Rybníček et al., 2010; Ge et al., 2013; Čermák et al., 2021). Under ongoing climate changes, more frequent and severe drought periods are expected (Williams et al., 2013). In fact, several extreme droughts and heat waves occurred in the last 25 years, including the studied year of 2015, which was one of the worst droughts recorded in Central and Eastern Europe (Van Lanen et al., 2016; Stojanović et al., 2017b). There is growing evidence that increasing climatic pressure on forest ecosystems, especially drought-related, causes widespread Norway spruce decline. During the drought period, the shallow root system of Norway spruce cannot meet the demand of the transpiring crown (Puhe, 2003), which substantially reduces growth (Nilsson and Wiklund, 1992; Altman et al., 2017) and makes it more susceptible to other abiotic and biotic stressors (Lindberg and Johansson, 1992; Økland and Berryman, 2004; Lindner et al., 2010). Consequently, this tree species exhibited the most severe vitality and growth problems in the last decades (Rybníček et al., 2010). Many authors predict that Norway spruce cultivation is likely to become risky and unprofitable in forests at low altitudes in Central Europe (Čermák et al., 2021; Krejza et al., 2021). In the Czech Republic, the original distribution of Norway spruce is related to annual precipitation of around 800 mm (with growing season precipitation in the range of 490-580 mm). According to Čermák et al. (2021), for the Czech Republic, areas with an annual temperature under 6.5°C with average annual rainfall above 700 mm are climatically suitable for the secure cultivation of Norway spruce. Krejza et al. (2021) estimated that the elevation of approximately 900 m a.s.l. is a threshold between the positive and negative growth response to warmer and drier conditions caused by changing climate. Understanding the response of Norway’s spruces to drought is crucial for assessing its acclimation potential under a more arid future climate.

Transpiration, the process of water movement through a plant and its evaporation into the atmosphere, is coupled to photosynthesis through stomata functioning (Jarvis and Davies, 1998). Transpiration or tree sap flow measurements can be particularly suitable for investigating the different trees’ strategies for coping with limited soil water availability (Zhang et al., 2018) and increasing evaporative demand (Nelson et al., 2020). Trees can regulate their stomatal aperture during drought periods to avoid excessive water loss and maintain the functionality of the hydraulic system *via* the prevention of xylem cavitation (Aranda et al., 2012). Consequently, prolonged stomatal closure causes a decline in photosynthesis rates and biomass accumulation (Poyatos et al., 2021). We can place a plant species on the isohydricity vs. anisohydricity spectrum based on preference towards xylem embolism avoidance or carbon starvation avoidance (Klein, 2014; Hartmann and Trumbore, 2016; Hochberg et al., 2018; Chen et al., 2021). Norway spruce exhibited in previous studies more isohydric stomatal behavior, thus minimizing the risk of hydraulic failure *via* timely stomatal closure and reduction of excessive water losses (Kurjak et al., 2012; Oberhuber et al., 2015; Matthews et al., 2018). Prolonged droughts under an isohydric strategy can lead to the depletion of carbohydrates reserves, which could have a negative impact on the recovery potential of Norway spruce (Tomasella et al., 2017; Hajek et al., 2022).

Measurements of stem radial growth are useful tools indicating the impact of drought on biomass accumulation and partitioning. The radial growth of Norway spruce has been impaired due to more frequent and severe drought episodes in Central Europe (Sedmáková et al., 2022). The negative impact of drought on the growth and vitality of Norway spruce is further amplified by co-occurring heatwaves (Bosela et al., 2021). The ecological stability (defined as ability of ecosystem to self-regulate to return to steady state after perturbations (Rutledge et al., 1976)) of Norway spruce monocultural plantations outside its natural range has been significantly worsened in recent decades (Kunert, 2020; Krejza et al., 2021). Moreover, according to predictions of climate change models, we can expect further growth disturbances of Norway spruce driven by drought stress (Treml et al., 2022). In addition to stem radial growth dynamics, dendrometers allow us to monitor tree water balance by observing diurnal fluctuations in stem circumference caused by dehydration (shrinking) and rehydration (swelling) (Ježík et al., 2015; Szatniewska et al., 2022). Dendrometers are often used to evaluate tree water status by calculating Tree Water Deficit (TWD) (Drew and Downes, 2009; Zweifel, 2016). Dendrometer-based assessments of TWD correlate with the measurements of plant water status, such as leaf water potential (Ehrenberger et al., 2012). Trees can use the stem water reserves to maintain transpiration under short-term water deficit, which is also reflected in TWD dynamics (Čermák et al., 2007; Preisler et al., 2022). Combining tree sap flow measurements with radial growth dynamics (including TWD as a derived quantity) on trees in different soil water regimes brings valuable information about the behavior of Norway spruce trees under drought stress.

In this study, we evaluated tree sap flow, tree water deficit, and stem radial increment in ambient precipitation and under reduced rain throughfall during two growing seasons with contrasting hydro-climatic conditions (extremely dry year and year after drought with more favorable precipitation distribution). An experimental drought caused by throughfall exclusion coincided with a severe drought event in 2015, allowing us to observe how forests that experienced long-term water limitations would respond to extreme droughts. We hypothesize that trees growing under reduced precipitation would show a more conservative water use and lower radial growth in response to drought and, consequently, lower recovery ability upon drought release.

## 2 Material and methods

### 2.1 Study site

**Figure1:**
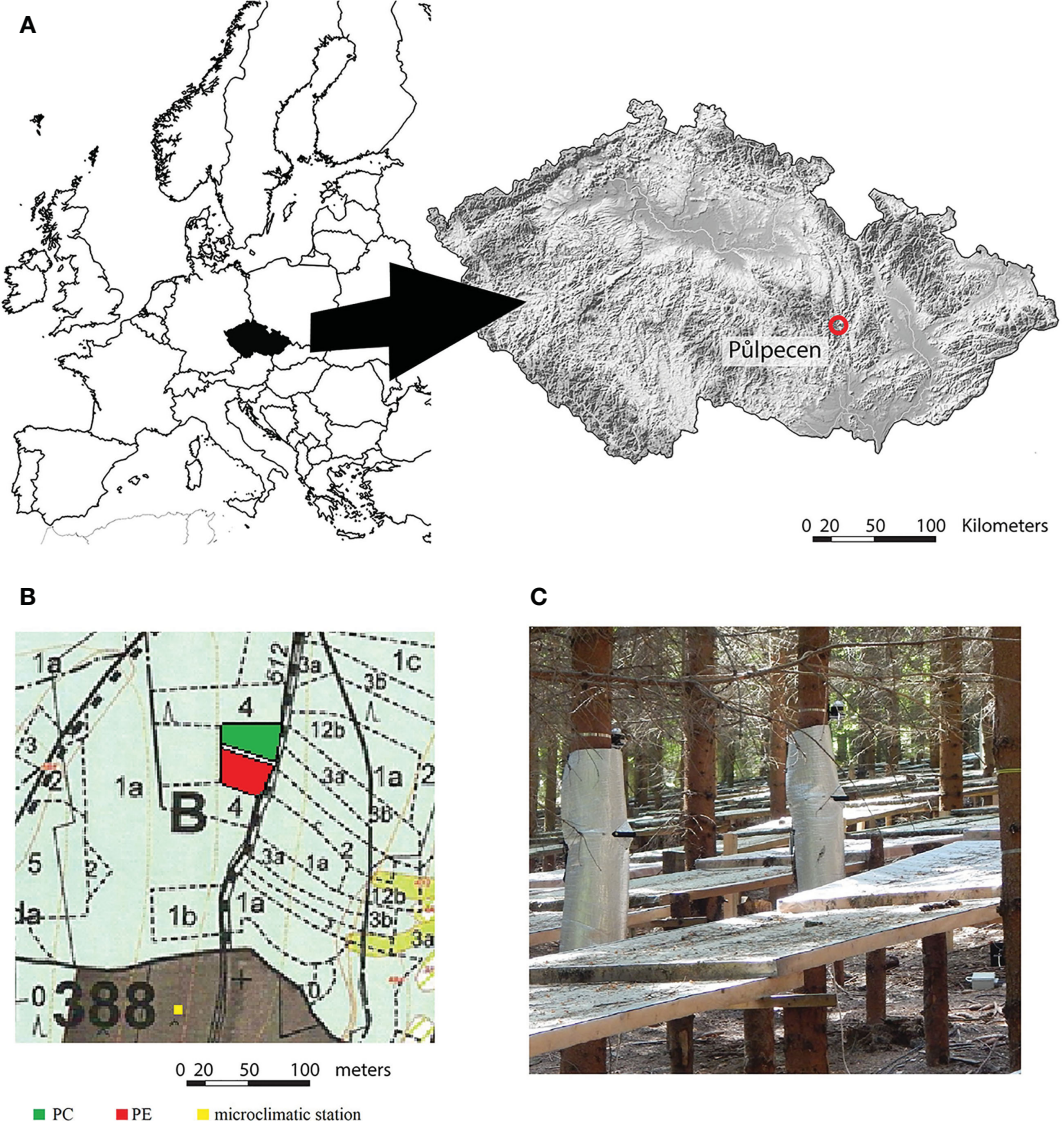
Location of the study **(A)**, location of plots (treatments) and microclimatic station (green plot: control (PC); red plot: precipitation exclusion (PE); yellow square: microclimatic station; light green: forest area; grey: open field) **(B)**, and the construction for rainfall exclusion in PE **(C)**.

The study was conducted near Půlpecen in the Czech Republic (N 49°36´38´´, E 16°31´39´´; 440 m a.s.l., EES exposition, 6-10° slope) (**Figure 1A**), and consisted of two plots within same stand of monocultural Norway spruce ~ 30 years old (**Figure 1B** and **Table 1**). The location of the stand at low altitude is assessed as not suitable for secure cultivation of Norway spruce, especially as a dominant species (Čermák et al., 2021; Krejza et al., 2021). In the plot with precipitation exclusion (hereafter PE), the space between trees was covered by plastic foil roofs installed 1 meter above the ground (**Figure 1C**), the roofs were about 2 meters wide as was the space between tree rows, and they covered about 70% of the area (the space between trees grown in the rows was not covered). The rainfall flew downhill on the roof surface outside the plot. The construction restricted the infiltration of rain into the soil but allowed free air movement below the roofs and soil evaporation. The second plot represented the control (hereafter PC) with ambient conditions. To estimate, what was the magnitude of precipitation exclusion, we collected precipitation throughfall by the set of three-meter-long gutters collecting water into the barrels, four per treatment. The collected water was measured every two weeks during growing seasons. Based on these measurements, we estimated that about 25% of rain throughfall was excluded in PE compared to PC. PE was established in 2007. Thus, trees in the PE had grown for seven years with reduced water availability before the primary measurements of this study. Plots did not differ in the distribution of tree stem diameter at 1.3 m (DBH) (**Supplementary Figure 1A**).

**Table 1:** Characteristics of studied sites with treatments.

Location	Půlpecen, Březova nad Svitavou, Czech Republic	
Mean annual temperature	7.2°C*, 7.9°C**	
Mean annual precipitation	711 mm*, 702 mm**	
Soil type	luvic Cambisol formed on the calcareous clayish sandstone, 120 cm deep	
Forest type***	4B2 forest type - beech zone with nutrient-rich soils - climatic optimum for *Fagus sylvatica*	
Plot (treatment)	PC – control plot	PE – precipitation exclusion
Plot area [m^2^]	825	1056
Stand density [tree ha^-1^]	1455	1306
Stand basal area [m^2^ ha^-1^]	29.3	27.2
Mean DBH (SD) [cm] (2014)	15.6 ( ± 3.9)	15.8 ( ± 4.1)
Mean H (SD) [m] (2014)	15.1 ( ± 3.6)	15.3 ( ± 3.7)

*normal period: 1961-1990, **normal period 1981-2010 (source: CHMI), ***Viewegh et al. (2003).

### 2.2 Microclimatic conditions

Global radiation (GR, [MJ m^-2^ day^-1^]), air temperature (T_air_, [°C]) and relative air humidity (RH, [%]) were measured at a one-hour interval (Minikin RTHi, EMS Brno, Czech Republic), at the open field in 200 meters to measured stand (**Figure 1B**). Data were used for calculating daily average (VPD [Pa]) and daily potential evapotranspiration (PET, [mm]) following Allen et al. (1998). Daily precipitation data (P, [mm]) were derived from the nearby meteorological station in Březová nad Svitavou, 8 km away. In 2016 soil water potential (SWP [bar]) was measured using gypsum blocks GB2 (EMS Brno, Czech Republic) once per hour at three different depths (10, 25 and 40 cm), on two sites per treatment. It is important to note that the measuring range of the gypsum blocks is 0 - -15 bar (SWP value generally referred as permanent wilting point (Hsiao, 1973; Šrámek et al., 2019), so all SWP records below this value are marked as -15 bar. In 2015, we conducted the measurements of soil water content (SWC, [%]) within the first 15 cm using (TRIME-PICO 32 Probes, IMKO Micromodultechnik, Germany), weekly on 20 points in PE (including under foil cover and between trees in rows, without cover) and 20 points in PC. SWC measurements were then compared with the same set up of measurements from the nearby site, with similar soil conditions and applied treatment (control plot and precipitation exclusion with roofs). The results of SWC were comparable, therefore, we justified the application to use SWP data from the other plots for our site. To evaluate differences in water availability between the years, we calculated cumulative water deficit (CWD), following Čermák and Prax (2009), defined as the difference between cumulative precipitation and cumulative potential evapotranspiration.

### 2.3 Sap flow

Sap flow was measured on ten trees per treatment during two consecutive growing seasons, 2015-2016. Measurements covered almost the entire two growing seasons (30.4-2.10 in 2015, and 21.4-17.10 in 2016). The tissue heat balance method (THB) (Čermák et al., 2004) was used to measure sap flow using the EMS 51 sensors (EMS Brno, Czech Republic) installed on the sampled trees at 1.5 m above the ground. Measurements were conducted once each minute, with average data recorded in ten minutes intervals. Original data were processed in the Mini32 software (EMS Brno, Czech Republic) to eliminate the heat dissipation loss by creating a baseline from the night-time zero-flow values under null evaporative demand. Specific sap flow (Q) was expressed as the mass of water passing through one centimeter of stem circumference over time [kg h^-1^ cm^-1^]. Sample tree selection was made according to Čermák et al. (2004) “quantile of total” method, based on an annual stand inventory of DBH of all trees. We calculated cumulative basal area and DBH for sample trees so they will reflect the stand structure (**Supplementary Figure 1B**). We chose trees to represent the same DBH classes in both studied plots, which minimizes the tree size effect on transpiration. Moreover, there were no significant differences between treatments in size (DBH) of the sampled trees (*p* = 0.887). The mean DBH (SD) of sample trees was 19.4 (3.3) cm and 19.5 (3.3) cm in PC and PE, respectively.

### 2.4 Dendrometer and tree-ring measurements

Stem circumference changes were monitored using automatic band dendrometers (DRL 26, EMS Brno, Czech Republic) installed on the same trees as sap flow sensors, 2 m above the ground. The bark surface was smoothed to reduce the influence of hydroscopic shrinkage and swelling of the bark on the dendrometer readings (Zweifel and Häsler, 2001). Data were recorded and stored in the one-hour interval at a µm resolution. Stem circumference readings were transformed into stem radius variations (SRV, [µm]). Since the dendrometers measure not only stem growth but also reversible changes in tree water balance (shrinkage and swelling of elastic tissues due to de- and rehydration), stem growth and changes in the tree water status were calculated using the zero-line approach (GRO, [µm]) following Zweifel (2016). The zero-line approach used assumes that stem growth occurs only when the stem radius exceeds the previous maximum radius (Zweifel, 2016). The GRO line represents a one-directional process that only increases the radius of the stem. The differences between GRO and observed SRV values were considered a measure of tree water deficit (TWD, [µm]). Additionally, in autumn 2014 prior to sap flow and dendrometer installation, 10 trees per plot were cored by a Pressler increment borer. Two cores were taken per tree perpendicular to the maximum slope. We aimed here to test whether the trees from PE and PC plots experienced different long-term growth trends, by analyzing tree ring widths (TRW, [mm]). The wooden cores were prepared, measured and cross-dated as previously described in detail by Stojanović et al. (2017a). TRW was measured to the nearest 0.01 mm and tested for the differences between treatments in each year separately.

### 2.5 Data analysis

The statistical analyses and data visualization were performed using SigmaPlot (ver. 14.0; Systat Software Inc., Chicago, USA) and R statistical software (ver. 4.2; R Core Team, Vienna, Austria). The level of significance in all tests was set at *p* < 0.05. The maximal daily averages were calculated as averages from the maximal daily Q of all sampled trees for each year and treatment. The data were checked for temporal autocorrelation (identified in Q and TWD) which were considered in the following analyses. Temporal autocorrelation is the way that one variable relates too much to itself in the events occurring in the small subsequent time step. For daily Q and TWD, we assessed trend and seasonal analysis by the Kwiatkowski-Phillips-Schmidt-Shin (KPSS) test and the Ljung-box test, respectively. Autoregressive integrated moving averages (ARIMA, Ripley, 2002) were fit to the daily Q and TWD time series using an iterative Box-Jenkins approach where autocorrelation and partial autocorrelation were interrogated and accepted only when detrended models without autocorrelation in residuals were confirmed. ARIMA is the model that explains a given variable based on its own previous values (lags). Autocorrelation and partial autocorrelation were checked visually by *acf* and *pacf* functions, accordingly (library *stats*, Venables and Ripley, 2002), (**Supplementary Figure 3**), and confirmed by the Breusch-Godfrey test (library *lmtest*, Johnston, 1984). These modeled data were used for the comparison of the daily datasets.

Moreover, the data were tested for normal distribution by Jarque-Bera test and explored by quantile-quantile graphs. The modeled Q values which did not meet normality prerequisites were transformed by logarithm. Differences in annual GRO and daily modeled values of Q and TWD between years and treatments were analyzed by two-way ANOVA and followed by a *post-hoc* Tukey test for statistical significance among the interactions. Comparison of daily TWD was performed by Analysis of Variance of Aligned Rank Transformed Data (ANOVA of ART, Wobbrock et al., 2011) also followed by the *post-hoc* Tukey test. The Student’s t-test was used to test TRW among the treatments in the years 1994-2014 with Hommel’s correction for *p*-values.

We used linear regression analysis to explore the impact of microclimatic conditions (VPD and GR) and water-soil conditions (SWP) on Q and TWD. The regression was completed with an iterative Cochrane-Orcutt transformation to overcome autocorrelation in the model’s residuals. The residuals were checked for autocorrelation by the Box-Jenkins test and Durbin-Watson Test, besides the visual graphs (**Supplementary Figures 4**-**6**). The transformed data were used for the modelling of the linear relationship. Mann-Whitney U Test tested differences in SWP between years and treatments and differences in VPD between the years.

## 3 Results

### 3.1 Microclimatic conditions

**Figure 2:**
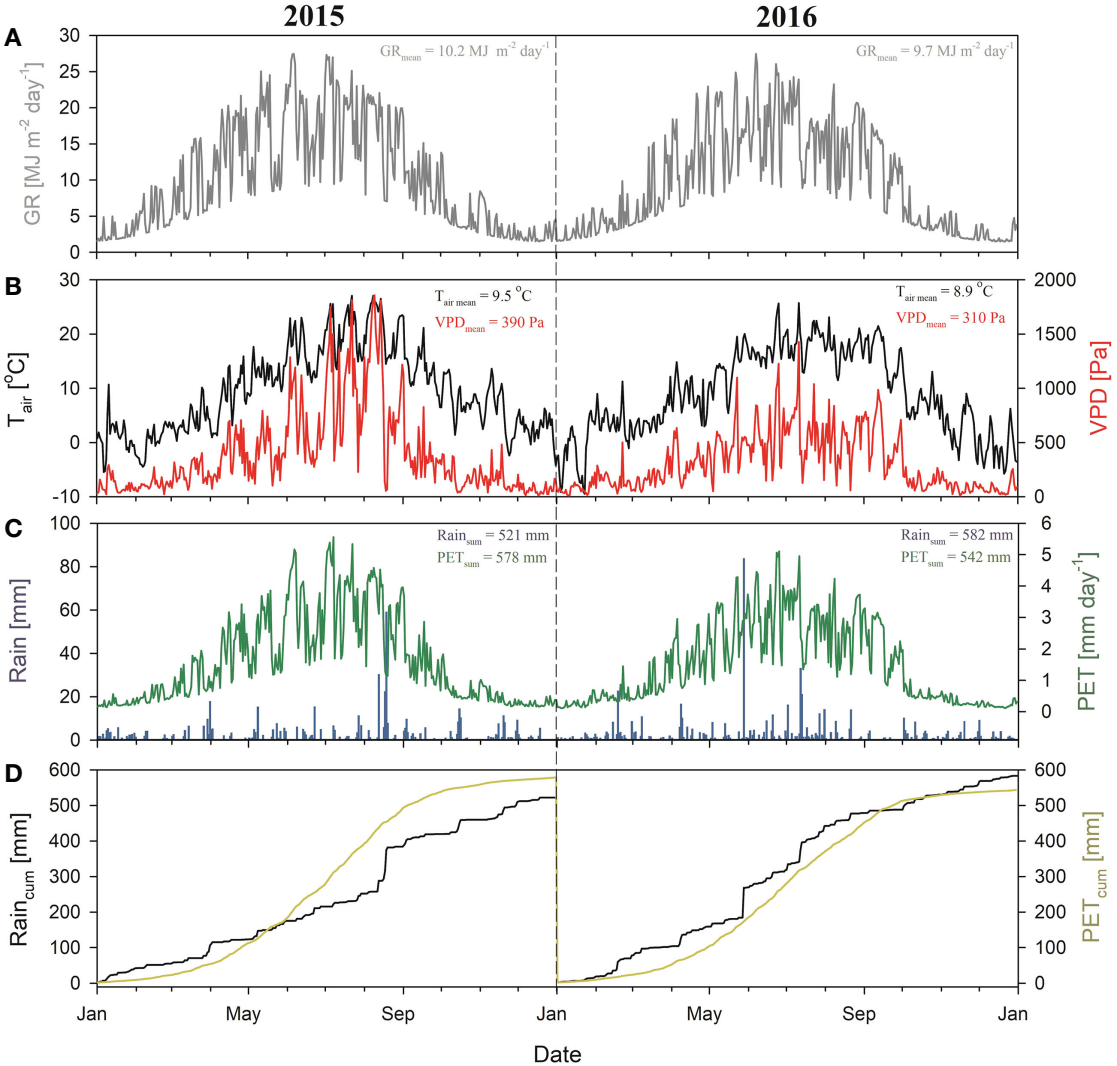
Microclimatic variables in 2015 and 2016 on the studied site. Daily sums of global radiation - GR **(A)**, daily mean air temperature - Tair and vapor pressure deficit - VPD **(B)**, daily precipitation - Rain and potential evapotranspiration - PET **(C)**, cumulative precipitation - Raincum and cumulative potential evapotranspiration - PETcum **(D)**. The difference between PETcum and Raincum is Cumulative Water Deficit (CWD).

**Figure 3:**
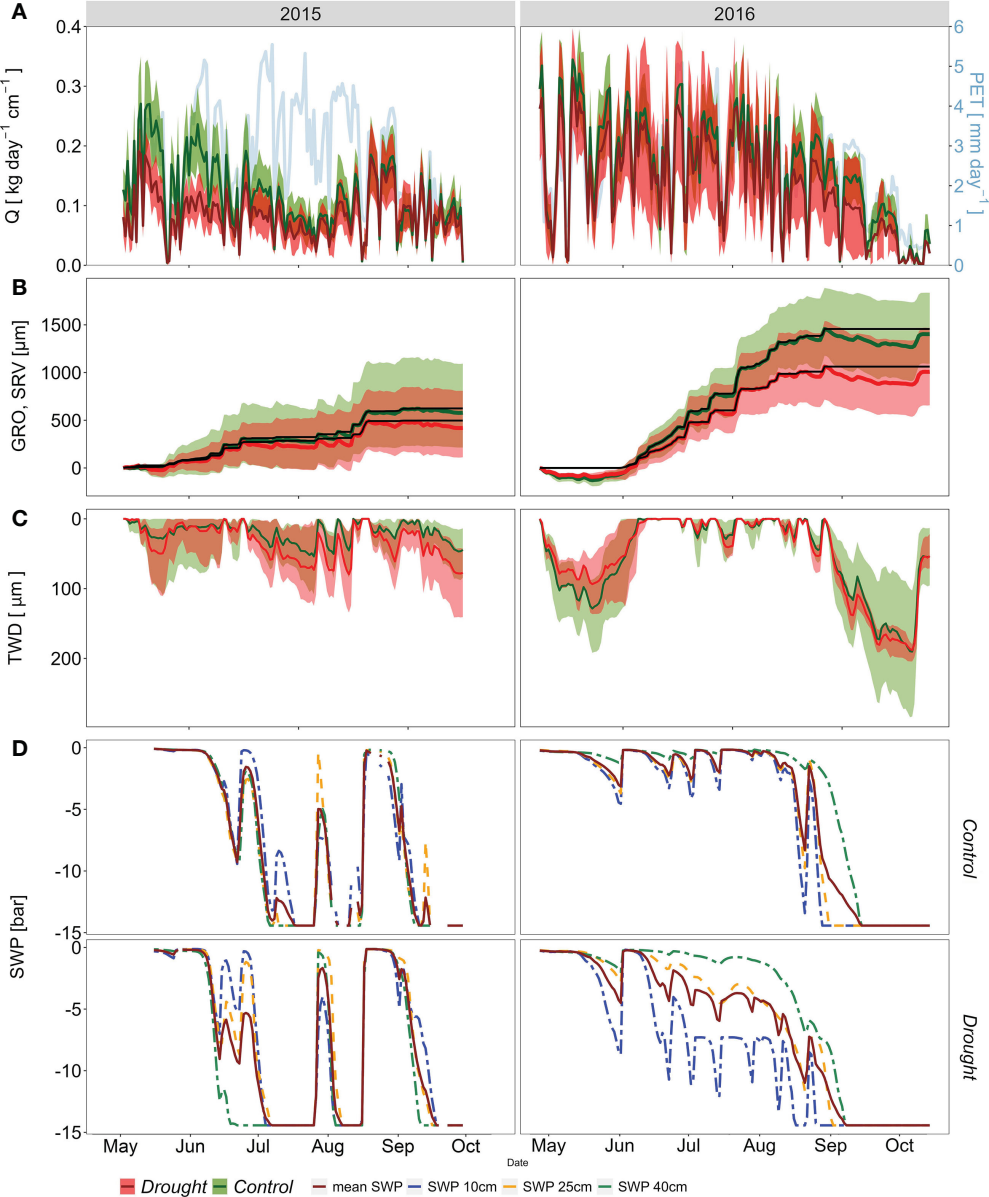
Seasonal dynamics of specific sap flow - Q and potential evapotranspiration - PET **(A)**, stem radial variation - SRV (bold colored line) and modelled growth line - GRO (black line) **(B)**, daily tree water deficit - TWD (in absolute values) **(C)**, and mean soil water potential – SWP and at depths: 10, 25 and 40 cm, in PC (upper) and PE (lower) **(D)** in the growing seasons: 2015 (left) and 2016 (right). The curves in A)-C) represent mean values ± confidence intervals.

The microclimatic conditions differed between the studied years (**Figure 2**), as well as in comparison to long-term climatic observations. Both the 2015 and 2016 years were significantly warmer (mean annual air temperature: 9.5°C and 8.9°C) and with lower precipitation (annual amount: 521 mm and 582 mm) than the long-term normal period means (Tab. 1), respectively. However, 2015 was a more arid year, with a higher annual average temperature and lower precipitation than in 2016. There were four distinctive heatwaves across the summer months in 2015 (~25 days), with maximum daily temperatures exceeding 30°C. The maximum value of CWD was reached in the middle of August (-175 mm) (**Figure 2D**). Moreover, in 2015, even in PC, 66 days had the mean SWP below the -12 bar (moderate water stress according to Hsiao (1973) from which 51 had SWP below -15 bar (permanent wilting point) (**Figure 3D**).

In 2016, annual PET was lower by 36 mm than the previous year. VPD as a key factor controlling evaporative demand was significantly lower in 2016 compared to 2015 (*p* < 0.01). Annual rainfall exceeded PET by 40 mm, and a maximum CWD of -25 mm was reached at the beginning of October after a dry September (**Figure 2D**). The mean SWP dropped below -12 bar only in the second half of August and September (**Figure 3D**). However, low values of SWP were then not associated with high VPD. Even though SWP values in PE were significantly lower than in PC in all measured soil depths (*p* < 0.05), they did not indicate water stress during the majority of the growing season.

### 3.2 Temporal dynamics of sap flow, tree water deficit and growth

**Figure 4:**
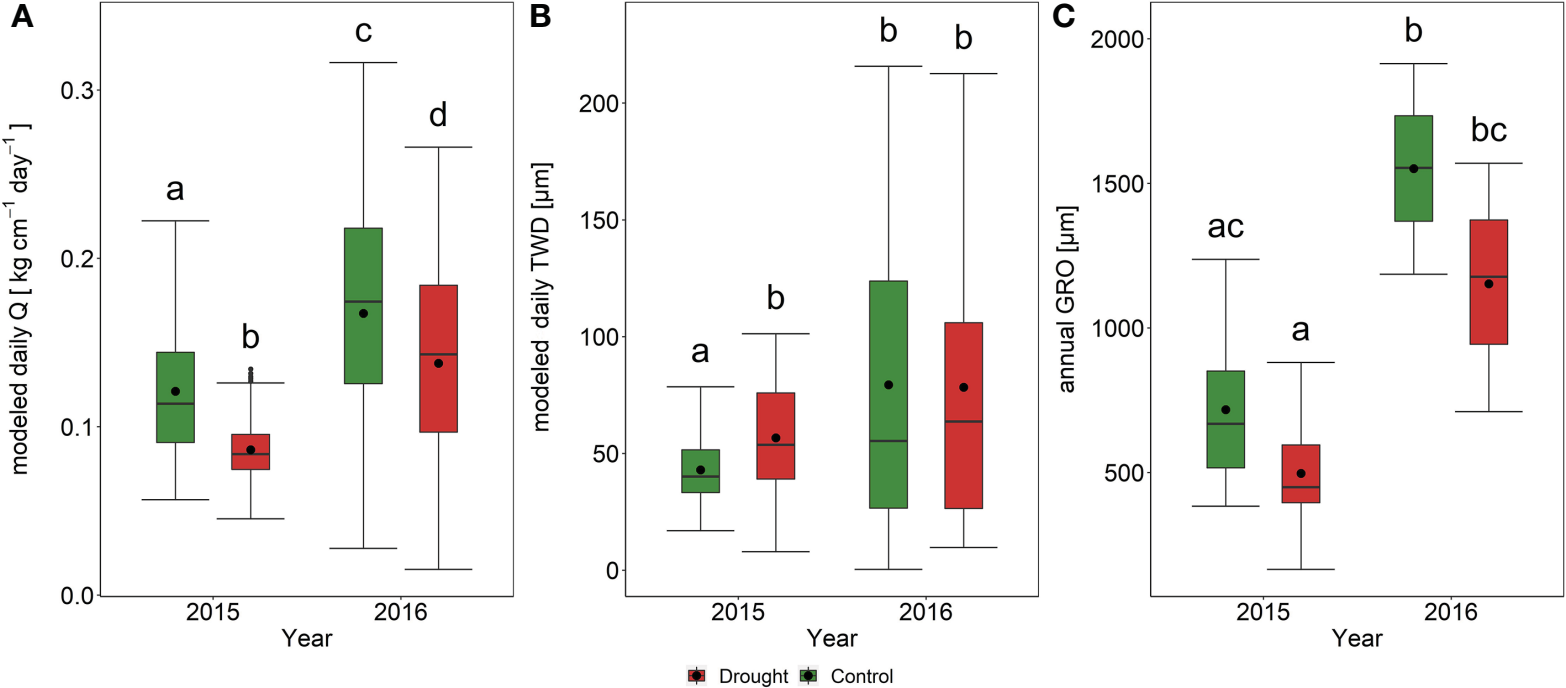
Boxplots for values of modelled specific sap flow - Q **(A)**, modelled tree water deficit - TWD **(B)**, and annual values of radial growth - GRO **(C)**. The data are expressed as medians (solid lines) and means (dots) of measured modeled values. The box boundaries mark the 25th and 75th percentiles, and the whiskers show the minimum and the maximum. Different letters indicate the statistically significant variation between the group means. The modeled values are derived from ARIMA model.

Contrasting hydro-climatic conditions in 2015 and 2016 were reflected in the dynamics of Q, SRV, and TWD among treatments (**Figure 3**). The two-way ANOVA results show that the main effect of the year was significant for daily Q and TWD values (**Table 2**). PE showed significantly lower Q during both years compared to the PC treatment (**Figure 4A**). The maximal daily average Q at PC was about 40% higher than that at PE in 2015. The seasonal course of Q in 2015 showed significant intra-annual variability. The most remarkable differences between PC and PE occurred during the spring, from May to the second half of June. Since then, soil moisture decreased rapidly, and trees in both plots reduced their Q to almost null values, even under very high evaporative demand (**Figure 3A**). For example, during the heat wave (16-26. July), when air temperature peaked over 34°C and VPD over 1800 Pa, Q decreased to 0.03-0.04 kg cm^-1^ day^-1^, in both PC and PE. Q increased after rain episodes in late August. However, PC and PE plots did not differ in Q until the end of the measurements. The precipitation seemed insufficient to enhance depleted soil water reservoirs, and the soil drought returned about two weeks later and remained until the end of the growing season. In 2016, the seasonal course of Q varied distinctively from the previous dry year, which is visible in higher maximal and average daily values (**Figure 3A**). During the 2016 season, the Q of PC trees started to diverge from PE trees from the second half of August. Afterwards, PC trees showed higher Q than PE trees till the point when mean soil water potential in both plots decreased below -12 bar (**Figures 3A, D**).

**Table 2:** Statistical test results for daily modeled specific sap flow (Q) and daily modeled tree water deficit (TWD) and measured annual radial growth.

Two-way ANOVA	Df	F stat	p-value
daily Q			
year	1	27650.79	<0.001
treatment	1	128.66	<0.001
interaction	1	57.37	<0.001
Two-way ANOVA of ART	daily TWD		
year	1	14.33	<0.001
treatment	1	3.34	ns
interaction	1	6.0	<0.05
Two-way ANOVA	annual radial growth		
year	1	25.396	<0.001
treatment	1	4.097	=0.052
interaction	1	0.363	ns

The ns means non-statistically significant.

Df refers to degrees of freedom, p-value to significance of the test, F stat to F statistics, two-way ANOVA to Analysis of Variance, two-way ANOVA of ART to Analysis of Variance of Aligned Rank Transformed Data.

In 2015 stem radial growth (GRO) started in mid-May (**Figure 3B**); however, it was soon inhibited by a hot drought spell that began in June and continued until mid-August (**Figures 2**, **3D**). Due to increased precipitation and subsequent improvement in soil water availability in August, the GRO reactivated only for a brief period until the beginning of September. On the other hand, in 2016, the GRO was initiated about 15 days later than in 2015; however, favorable conditions in terms of temperature and soil water availability (**Figures 2**, **3D**) were good preconditions for a successful GRO. Total annual radial growth was significantly higher in 2016 compared to 2015. However, there were no significant differences in GRO among the treatments in both years studied (**Figure 4C**). TRW chronologies revealed highly similar growth patterns among treatments (**Supplementary Figure 2**). The control and drought plot chronologies were highly correlated between them (Pearson’s *r* = 0.957, *p* < 0.0001). However, since 2010 mean tree-ring width was significantly higher (*p* < 0.05) in the trees sampled within the control plot, compared to those trees coming from the drought plot (**Supplementary Figure 2**).

Tree water deficit (TWD) significantly differed among the plots in 2015, while no differences were observed in 2016 (**Figure 4B**). TWD was distinct between observed years, with significantly higher values in 2016. Higher TWD values generally occurred in periods with soil water stress (**Figures 3C, D**). These negative values corresponded to the early spring and late summer drought in 2016. The first major decrease in TWD values early in the season was caused by high air evaporative demand during sufficient soil water availability, possibly causing later growth onset that year (**Figures 3B, C**). At the end of the growing season of 2016, TWD seemed to be enhanced by both relatively high evaporative demand and low SWP. Unexpectedly, TWD during 2015 showed a smaller amplitude and variability of values than in 2016, despite the year 2015 being markedly drier with higher evaporative demand (**Figure 2**).

### 3.3 Response of sap flow and tree water deficit to environmental conditions

**Figure 5:**
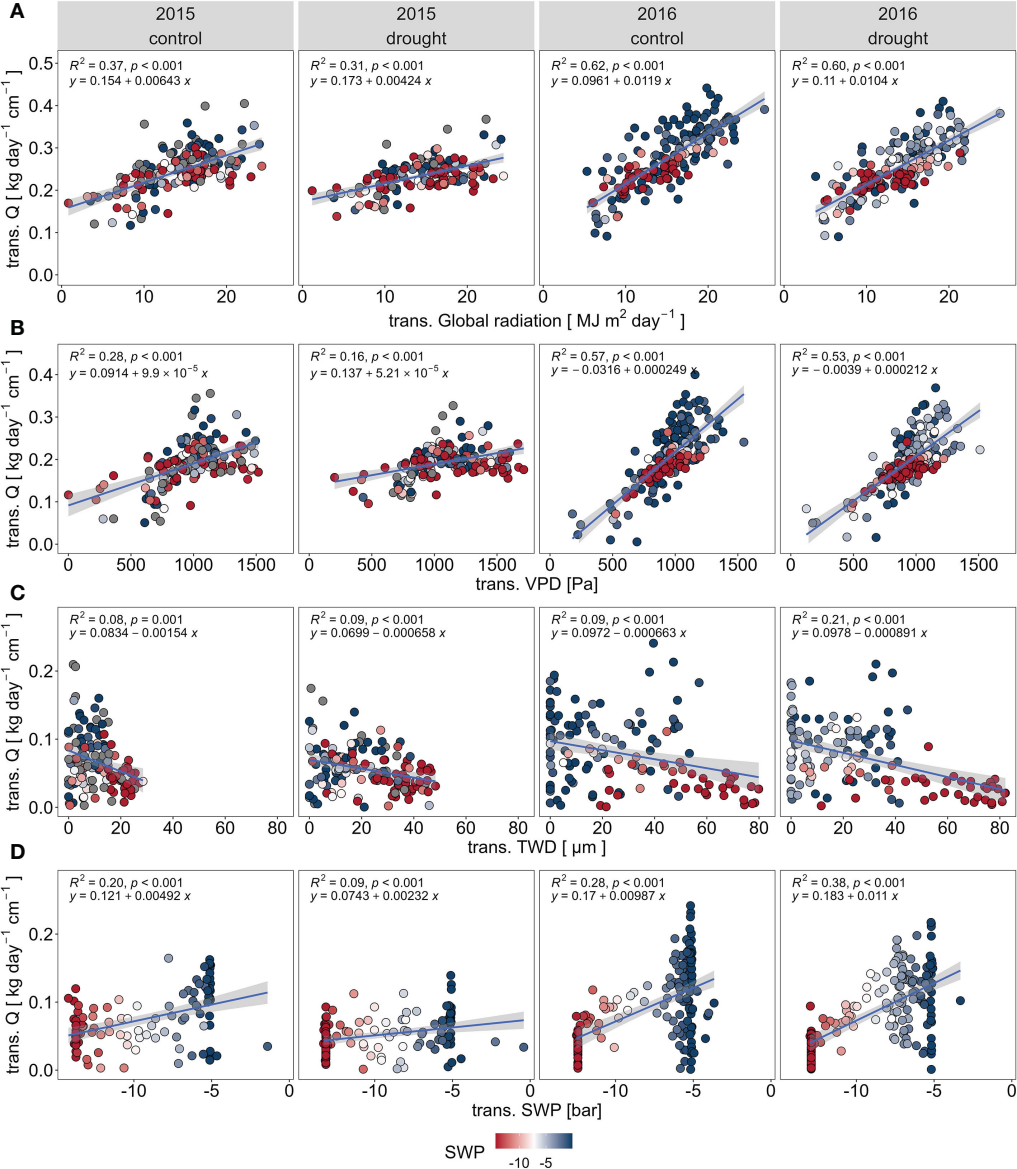
Responses of transformed specific sap flow Q to microclimatic conditions and transformed tree water deficit TWD in control (PC) and drought (PE) plots in 2015 and 2016: transformed global radiation GR **(A)**, transformed vapor pressure deficit (VPD) **(B)**, transformed tree water deficit TWD **(C)**, and transformed mean soil water potential SWP **(D)**. The values are derived from the Cochrane-Orcutt transformation.

**Figure 6:**
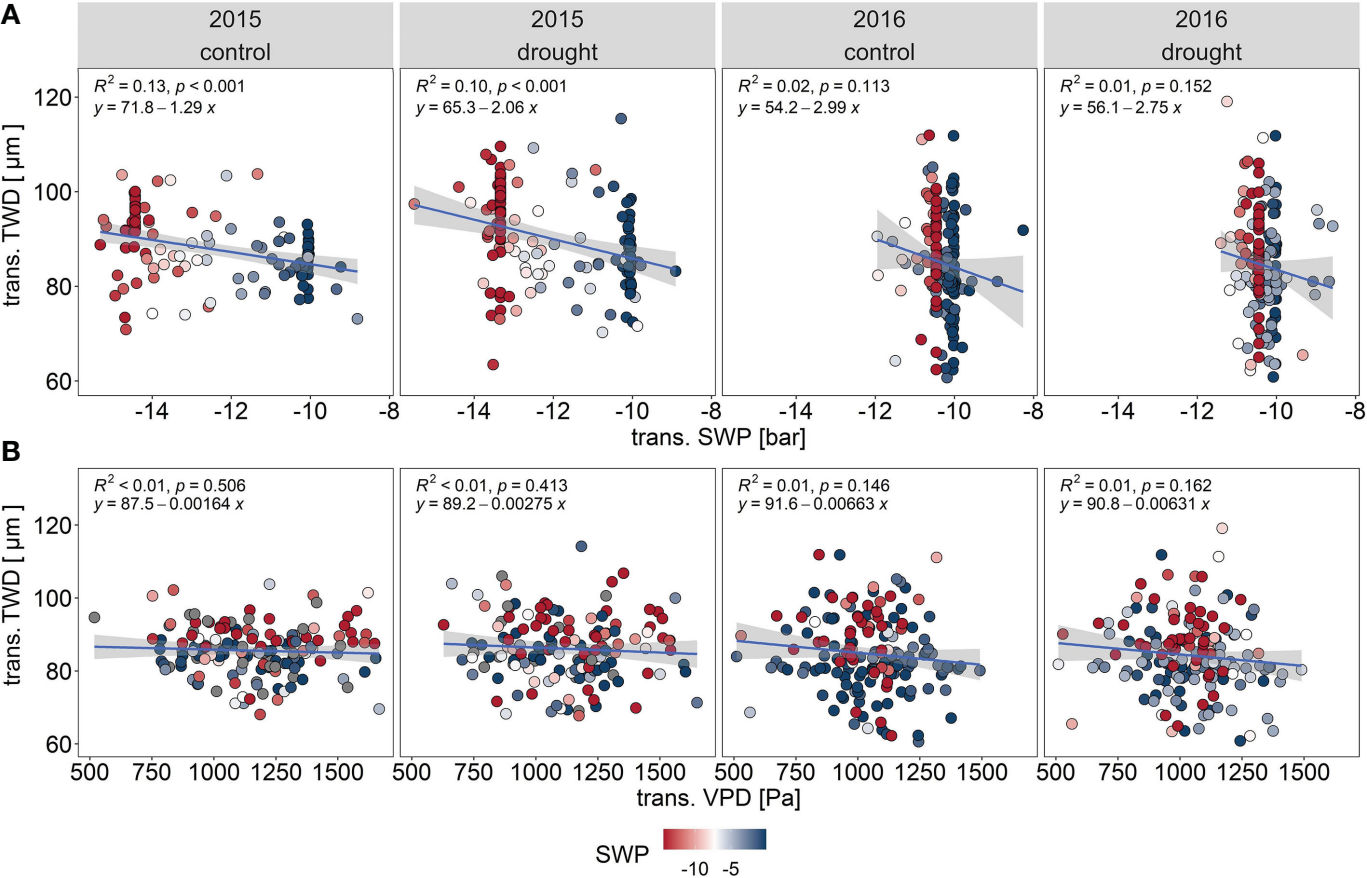
Responses of transformed tree water deficit TWD to microclimatic conditions in control (PC) and drought (PE) stands in 2015 and 2016: transformed mean soil water potential SWP **(A)**, and transformed vapor pressure deficit (VPD) **(B)**. The values are derived from the Cochrane-Orcutt transformation.

The slope of response of Q to GR and to VPD was steeper in the more the humid year 2016 than for the drier year 2015 for both treatments (**Figures 5A, B**). Moreover, the explained variability (R^2^) was also greater in 2016 than 2015. Norway spruce in both treatments showed more isohydric behavior during the drier year 2015, with a reduction of Q under high evaporative demand (**Figures 5A, B**). The relationship between Q and TWD was significant for both years and treatments, but with extremely low explained variability of Q by TWD values (**Figure 5C**). Decreasing SWP led to a reduction of Q in both treatments during both 2015 and 2016 (**Figure 5D**). TWD showed a low negative relationship with SWP in 2015, thus dropping SWP enhances TWD of Norway spruce (**Figure 6A**). VPD did not have significant impact on TWD in both years (**Figure 6B**).

## 4 Discussion

The summer of 2015 was the hottest and climatologically driest summer over the period 1950-2016, mainly affecting Central and Eastern Europe (Ionita et al., 2017). For the Czech Republic, it was the second-driest summer since 1950, whereas only 2003 had lower precipitation (Van Lanen et al., 2016). The environmental impact on the water deficit during 2015 was similar as the PC and PE treatments exhibited very similar Q, TWD and growth responses during this year. Nevertheless, the PE treatment showed significantly lower maximal Q. The recovery of Norway spruce after 2015 did not differ between the treatments, which means that either PC suffered similar legacy effect as PE or treatment effect (precipitation exclusion) on growth was not statistically significant.

### 4.1 Sap flow and isohydric behavior of Norway spruce

The PE treatment showed significantly lower maximal sap flow values than PC treatment in year 2015. This can be attributed to either lower xylem conductivity or reduced crown transpiration. Reduction of conduit area of PE could reflect the lower maximal sap flow (Fassio et al., 2009). Trees from PE treatment with smaller tracheid diameter and smaller conduit area would be theoretically more resistant to xylem embolism (Lovisolo and Schubert, 1998; Rosell et al., 2017; Levionnois et al., 2021). The PE treatment could also develop thicker pit membranes which would similarly improve the embolism resistance, but could have negative effect on hydraulic conductivity and therefore sap flow (Held et al., 2021; Kaack et al., 2021). As we do not see significant reduction in wood growth, based on stability of Huber value (0.3 for 30 years old Norway spruce based on Köstner et al., 2002), we could assume that the total leaf area is unchanged between treatments (Lusk et al., 2007; Mencuccini et al., 2019). The observed reduction of sap flow *via* lower total crown transpiration could be then theoretically driven by altered stomatal conductance and not leaf area. As we do not have gas-exchange measurements or xylem anatomy data, the reasons behind the maximal sap flow reduction in PE are just theoretical, but plausible. Both treatments showed relatively more isohydric behavior during the drier year 2015, than 2016. Nevertheless, PE treatment reduced its Q rates sooner than PC under decreasing SWP, probably due to the long-term priming effect of reduced water availability (Tombesi et al., 2018). In general, Q and transpiration rates are driven by ambient atmospheric conditions (VPD, GR, T_air_), creating evaporation demand (Cruiziat et al., 2002; Nalevanková et al., 2018). However, high VPD accelerates sap flow, particularly under sufficient soil water content (Matejka et al., 2009; Flo et al., 2021). In 2016, when soil water was not severely limited, sap flow followed patterns of VPD and GR linearly in both plots. But, in the advancing drought in 2015, sap flow did not respond proportionally to changes in evaporation conditions. The reason for the decreasing response of sap flow to meteorological parameters under drought can be stomatal closure to prevent water loss (Střelcová et al., 2013). As we do not have leaf gas-exchange data, this is nevertheless an assumption that could describe the observed dynamics of sap flow. Under advancing drought, soil water availability becomes the main limiting factor of sap flow (Střelcová et al., 2013; Vido et al., 2016). The substantial reduction of Q during the prolonged drought in 2015 corresponded to isohydric behavior of Norway spruce, described by many studies (Kurjak et al., 2012; Oberhuber et al., 2015; Matthews et al., 2018). Norway spruce can maintain a steady leaf water status over a wide range of evaporative demand and soil water deficit and keep stomata closed during hot summer (Zweifel et al., 2009). Norway spruce can synthesize abscisic acid to maintain stomatal closure under long-term drought (Pashkovskiy et al., 2019). This strategy prevents plants from early xylem embolization under drought (Matthews et al., 2018). However, limiting transpiration comes with the cost of restricting CO_2_ uptake and reduction of photosynthesis (Breda et al., 2006),which leads to the depletion of carbon resources and even to a reduction of the synthesis of defense compounds (such as resin, terpenes and phenols) (McDowell et al., 2008). We can see also in our results that growth in year 2015 where in both treatments sap flow was significantly reduced, reflecting lack of carbohydrates needed for growth. This can increase the susceptibility of trees to biotic stress agents, which are currently viewed as the main drivers of forest disturbance of Norway spruce in Europe (Nardi et al., 2022; Netherer et al., 2022). Even if Norway spruce reduces the risk of hydraulic failure *via* stomatal closure, it might increase the chance of being attacked and overwhelmed by biotic stressors.

### 4.2 Stem shrinkage under drought

TWD is closely related to drought and is affected mainly by a combination of atmospheric and soil conditions (Ehrenberger et al., 2012; Köcher et al., 2013; Oberhuber et al., 2015; Zweifel, 2016). Several studies showcased that dendrometer-based TWD can be a great indicator of tree water status as it is connected to the use of stem water storage to support transpiration under water shortage (Zweifel, 2016; Brinkmann et al., 2019). This internal water storage mitigates the effect of drought. It protects the integrity of the hydraulic system against cavitation caused by large differences between water potential along the soil-plant-atmosphere-continuum (Čermák et al., 2007). However, if drought occurs over a long period, water storage will deplete, resulting in hydraulic failure, desiccation of elastic tissues and damage, eventually causing a tree to die (Salomón et al., 2022). We also observed higher values of TWD under drying soil; however, the magnitude of TWD did not correspond well with the severity of drought when the year 2015 and 2016 were compared. TWD in both years and treatments was driven primarily by soil water potential, which was also confirmed in other studies (Ježík et al., 2015; Szatniewska et al., 2022). The absolute values of TWD decreased after rainfall events, as water absorbed by roots refilled the stem water reserves (Offenthaler et al., 2001). The use of internal water storage reflected by capacitance is a significant factor affecting the sap flow variability of Norway spruce (Preisler et al., 2022). According to Čermák et al. (2007), water stored in certain trees’ xylem and phloem tissues can meet the transpiration demand for about a week. However, without sufficient refilling, internal water storage could not support transpiration for a longer period without the risk of xylem cavitation. It is possible that Norway spruce utilized stem water reserves for transpiration during the more humid year 2016, but not during the drier year 2015. For instance, TWD during drier 2015 showed a smaller amplitude than 2016, likely because of much lower sap flow (i.e. closed stomata) and lower requirements for stored trunk water (Salomón et al., 2022). This is reflected in the significant relationship between Q and TWD in 2016 but not in 2015. However, when stomata are closed in advancing lack of soil water, transpiration ceases, and elastic tissues are already dehydrated; thus, low soil water availability reduces capabilities to re-hydrate stem tissues (Turcotte et al., 2011), and TWD may no longer serve as an indicator of degrading tree water status. Similarly, Schäfer et al. (2018) observed in Norway spruce a decrease in TWD amplitude above 27°C with increasing drought. This anomaly was likely due to reduced transpiration rates driven by high evaporative demand and exhausted soil water reserves. Therefore, we presume that this was the main reason why the TWD was smaller during an extreme drought in 2015 than at the beginning and end of the season in 2016. The rise of TWD in May-June 2016 was most probably caused by using stem water storage for transpiration compared to the same period in 2015. It is notable to point out that also the long-term drought-stressed trees in PE were able to refill the stem water reserves without any delays, compared to PC treatment. The isohydric strategy of Norway spruce is thus probably successful for xylem embolism avoidance under drought stress.

### 4.3 Radial growth and recovery after drought

Seasonal growth dynamics are tightly linked to cambial activity and xylogenesis (Gryc et al., 2011; Ježík et al., 2015). Therefore, the effect of drought on biomass production depends on drought duration, intensity, and timing (e.g., occurrence early or at the end of the growing season) (Rötzer et al., 2017). In 2015, a drought occurred early in the season (middle of June), which was a period susceptible to xylogenesis. In our study, the timing of drought coincided with a period of maximal growth (cell division and enlargement (Gryc et al., 2011)) and had a more pronounced effect on the total radius growth. According to Jaworski (2019), Norway spruce in Central Europe typically starts radial growth in the middle of May, peaks in the middle of June and slows down at the end of July. Based on 8 years of studies across Czech Republic and Slovakia, Sitková et al. (2018) presented that the culmination of radial increment for Norway spruce usually occurs around the beginning-middle of June. Krejza et al. (2022) in a study aimed on carbon uptake and allocation in mature Norway spruce monoculture (located 30 km from our study location), showed the radial growth onset on old Norway spruce in the middle of May (128 DOY), ends in the beginning of September (249 DOY) with the highest rates of daily increment between the second half of May till the beginning of August (139 to 214 DOY), which agrees with our study. Stem radial growth was impeded in 2015, probably due to long-term stomatal closure limiting CO_2_ assimilation. McDowell et al. (2008) suggested that trees with more isohydric behavior are more prone to carbon starvation, especially during prolonged drought periods. Krejza et al. (2022) also noted much lower values of annual radial increment in 2015 compared with 2012-2017, which was almost 40% lower than usual. According to study of Huang et al. (2021) dealing with carbon allocation on Norway spruce seedlings, during periods of limited carbon assimilation caused by stomatal closure under drought, Norway spruce trees tend to prioritize the accumulation of carbon reserves over the investment of assimilates to growth. Also, Rötzer et al. (2017) have found a significant reduction in trees’ radial increment during the heavy drought of 2015, especially in rainfall exclusion treatment. Trees in our PE treatment were exposed to a higher water deficit since 2007 and were able to recover from 2015 and exhibited higher radial increment in the year 2016. Generally, in both years, stem radial increment was lower in both treatments compared with large-scale averages of Norway spruce in Central Europe (Rötzer et al., 2017; Sitková et al., 2018; Krejza et al., 2022). The TRW values from wood cores also revealed long-term decreasing trend of radial wood increment for both treatments during the experimental period (2007 – 2016), probably due to increasing water deficit in the region (Bosela et al., 2021). The introduction of precipitation exclusion treatment seems to create diversion of TRW between the two plots, especially visible from year 2013. That suggests long-term weakening trees grown at unfavorable sites at low altitudes, besides applied treatment of precipitation exclusion.

### 4.4 Concluding remarks

Approximately 30 years old Norway spruce trees located at a low altitude in a monocultural stand showed isohydric behavior under the exceptional drought of 2015, reflected in sap flow reduction under high evaporative demand. The precipitation exclusion treatment was primed for water-deficit stress since 2007 and exhibited lower maximal sap flow. Radial growth was markedly lower for both treatments under drought stress in 2015, but trees in both plots partially recovered in 2016 and showed increased radial growth. The recovery of precipitation exclusion treatment was less pronounced than the control treatment, however, not significantly. The growth reduction of studied plots indicates that Norway spruce forests in the Czech Republic are at a tipping point and the expected worsening of climate poses a severe threat to their existence at lower altitudes.

## Data availability statement

The raw data supporting the conclusions of this article will be made available by the authors, at reasonable request.

## Author contributions

JS, OM, RP and MS contributed to the conception and design of the study. JS and MS performed field work and data assessments. IZ conducted the data analysis with support from the JS, PP and MS. IZ and JS wrote the original draft. All authors contributed to the article and approved the submitted version.

## Supplementary material

Supplementary Figure 1 Stem diameter at 1.3 m (DBH) structure in the studied plots (red bars – precipitation exclusion plot (PE), green bars – control plot (PC). State at the end of 2014) **(A)**. Sample tree selection based on cumulative basal area in the stand and “quantile of total” method (Čermák et al., 2004) **(B)**.

Supplementary Figure 2 The tree-ring width chronologies at control (PC, green) and drought (PE, red) plot. The box boundaries mark the 25th and 75th percentiles, and the whiskers show the minimum and the maximum. Dots mark outliners. The asterisks at the bottom indicate significant difference (*p* < 0.05) among specific years based on the Student’s t-test. The shaded area represents the year when the rain exclusion experiment started (i.e., 2007).

Supplementary Figure 3 Temporal autocorrelation plots of Q **(A)** and TWD **(B)** before (left) and after (right) applied ARIMA.

Supplementary Figure 4 Autocorrelation of the models’ residua from the linear relationship of TWD with VPD **(A)** and TWD with SWP **(B)** before (left) and after (right) Cochrane-Orcutt transformation.

Supplementary Figure 5 Autocorrelation of the models’ residua from the linear relationship of Q with GR **(A)** and Q with VPD **(B)** before (left) and after (right) Cochrane-Orcutt transformation.

Supplementary Figure 6 Autocorrelation of the models’ residua from the linear relationship of Q with TWD **(A)** and Q with SWP **(B)** before (left) and after (right) Cochrane-Orcutt transformation.
