# Supplementary figures and images for: Sap flow and growth response of Norway spruce under long-term partial rainfall exclusion at low altitude
Source: Front Plant Sci. 2023 Feb 14;14:1089706. doi: 10.3389/fpls.2023.1089706 (PMC9974152; doi:10.3389/fpls.2023.1089706)

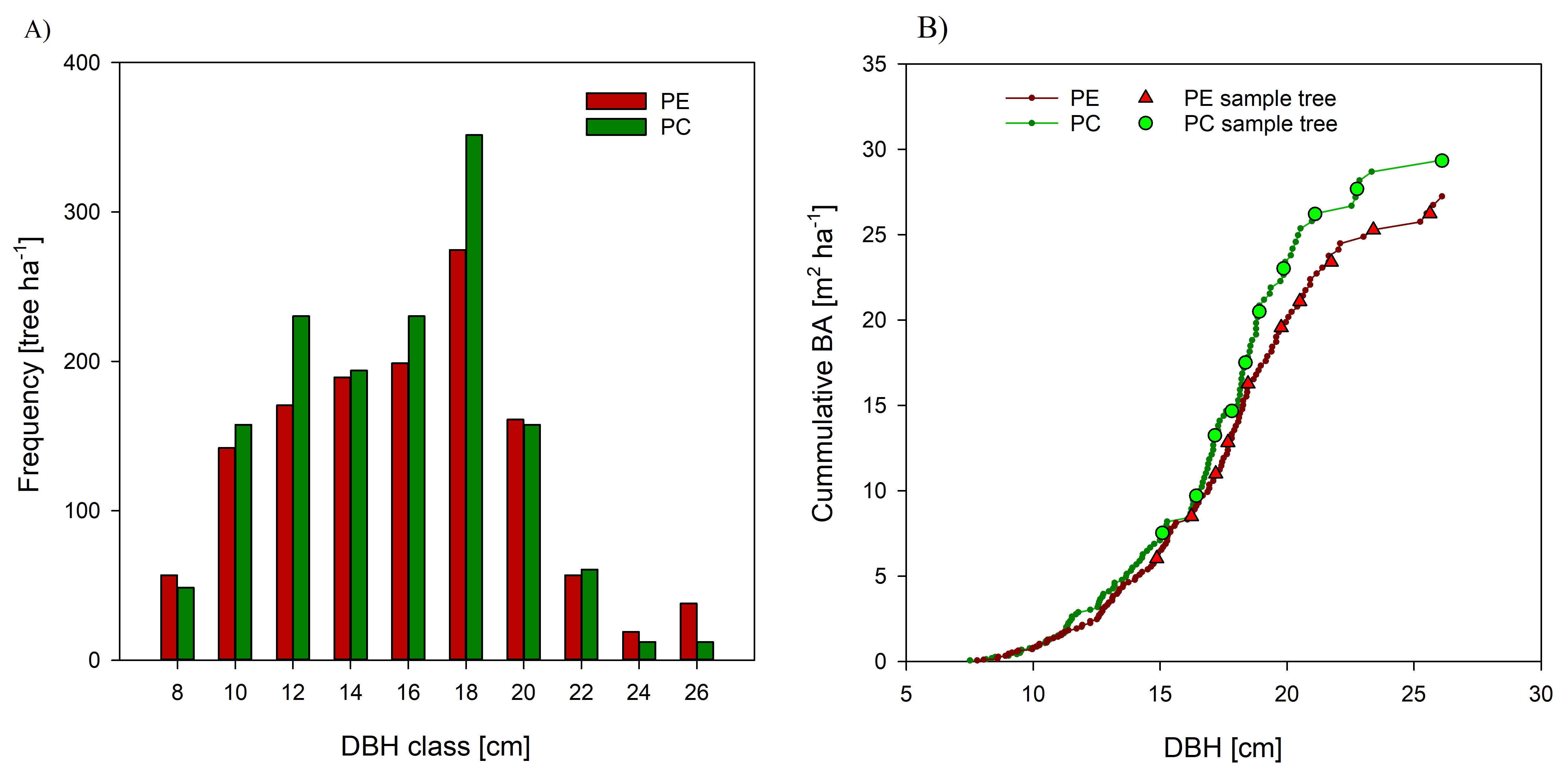

Supplement: Supplementary Figure 1 — Stem diameter at 1.3 m (DBH) structure in the studied plots (red bars – precipitation exclusion plot (PE), green bars – control plot (PC). State at the end of 2014) (A). Sample tree selection based on cumulative basal area in the stand and “quantile of total” method (Čermák et al., 2004) (B). [file Image_1.jpeg]

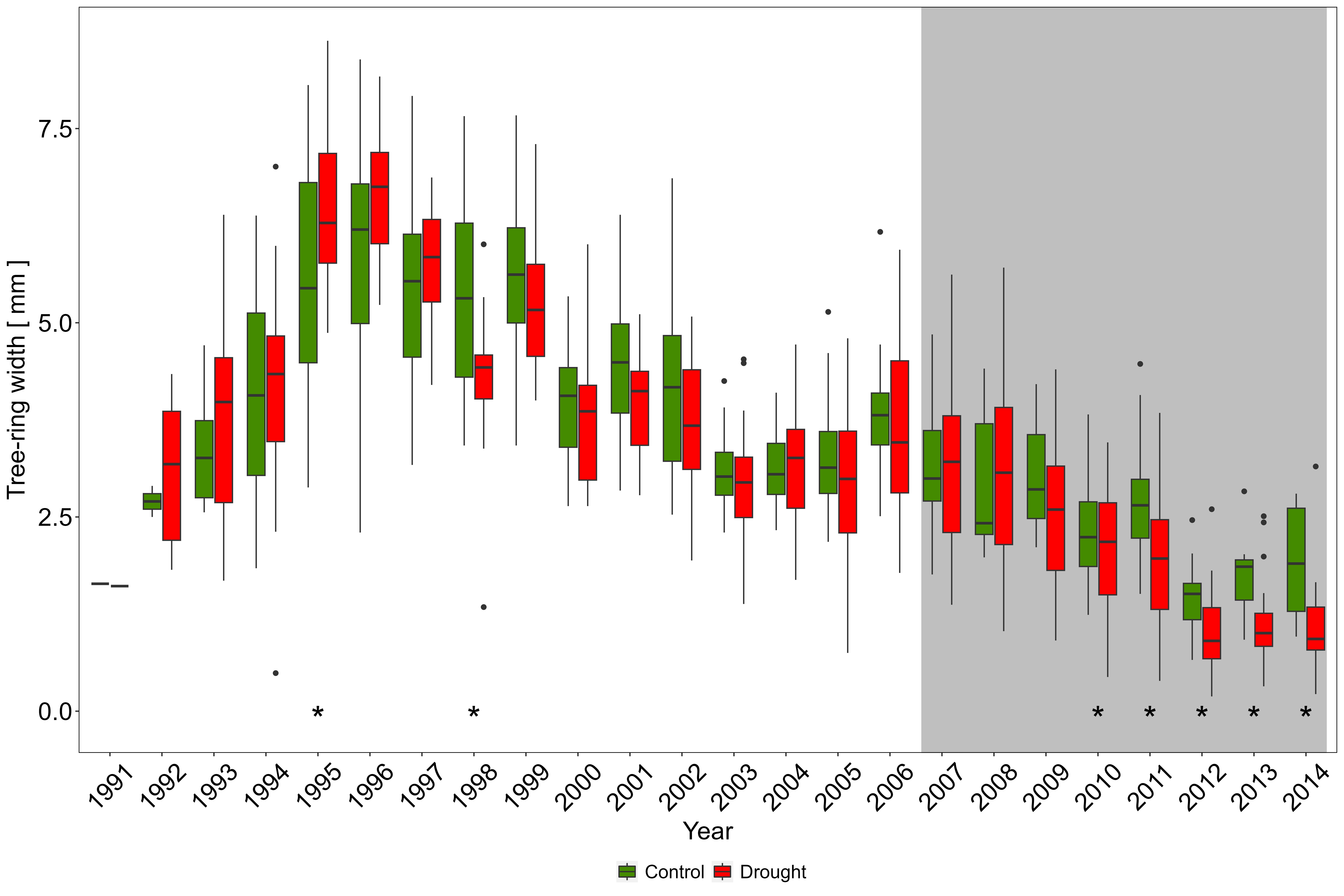

Supplement: Supplementary Figure 2 — The tree-ring width chronologies at control (PC, green) and drought (PE, red) plot. The box boundaries mark the 25th and 75th percentiles, and the whiskers show the minimum and the maximum. Dots mark outliners. The asterisks at the bottom indicate significant difference (p < 0.05) among specific years based on the Student’s t-test. The shaded area represents the year when the rain exclusion experiment started (i.e., 2007). [file Image_2.jpeg]

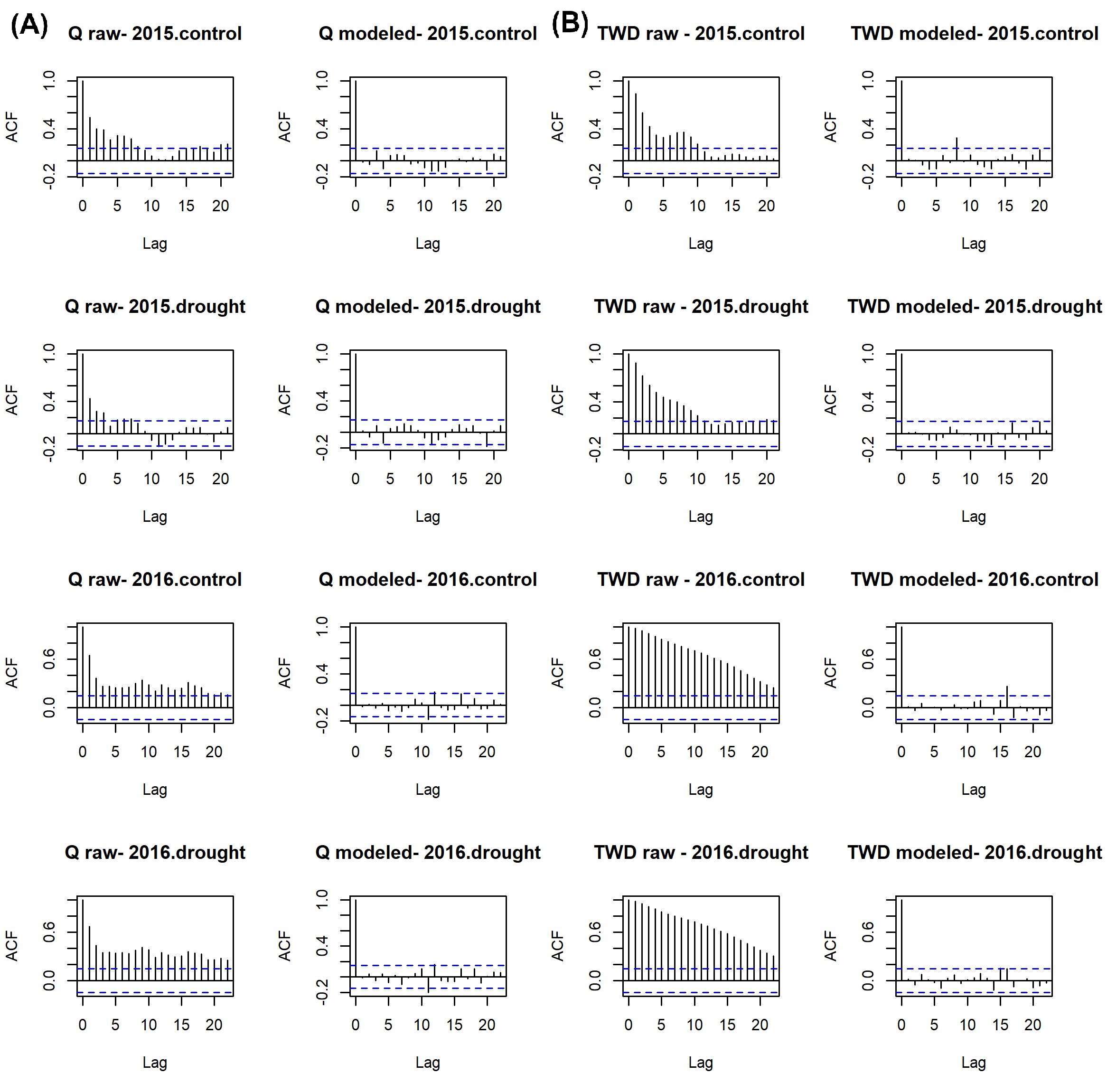

Supplement: Supplementary Figure 3 — Temporal autocorrelation plots of Q (A) and TWD (B) before (left) and after (right) applied ARIMA. [file Image_3.jpeg]

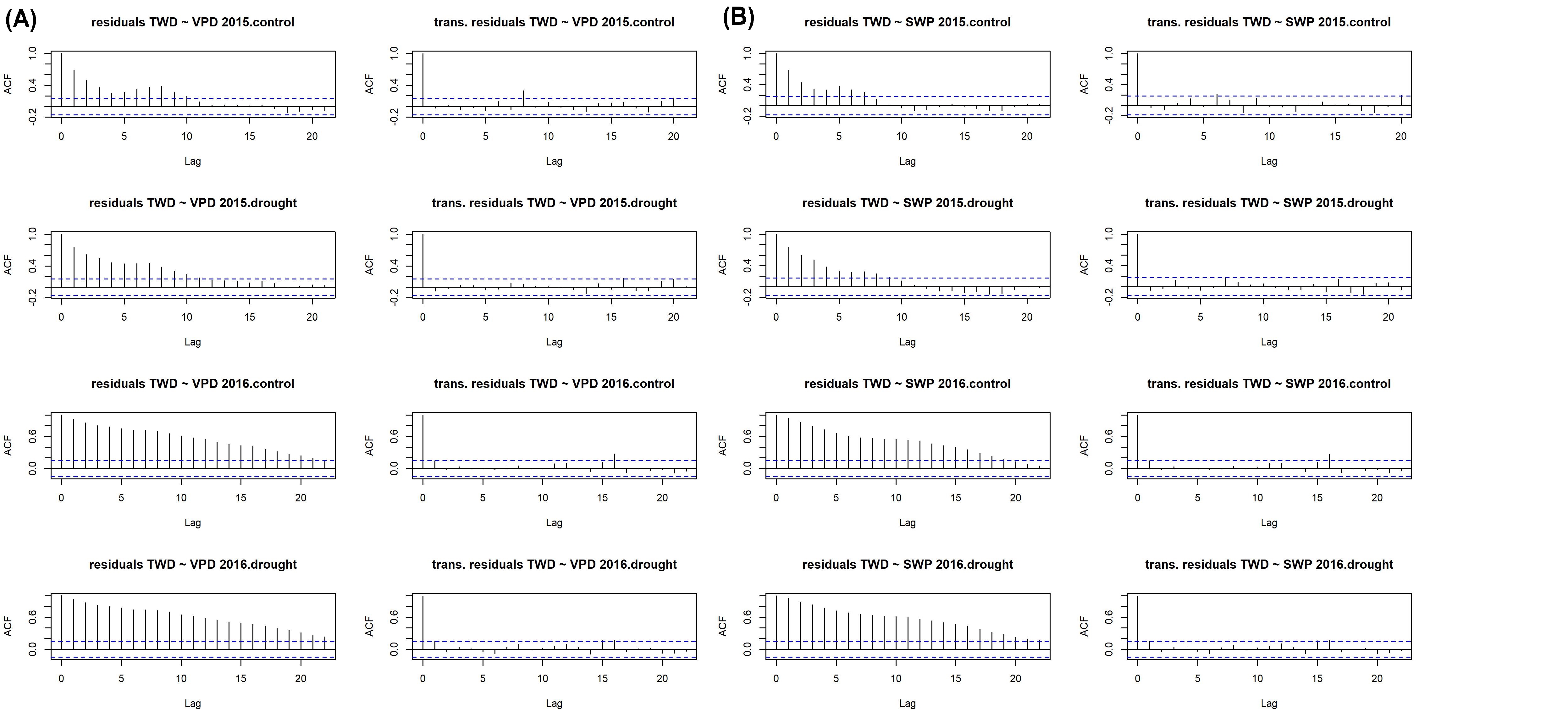

Supplement: Supplementary Figure 4 — Autocorrelation of the models’ residua from the linear relationship of TWD with VPD (A) and TWD with SWP (B) before (left) and after (right) Cochrane-Orcutt transformation. [file Image_4.jpeg]

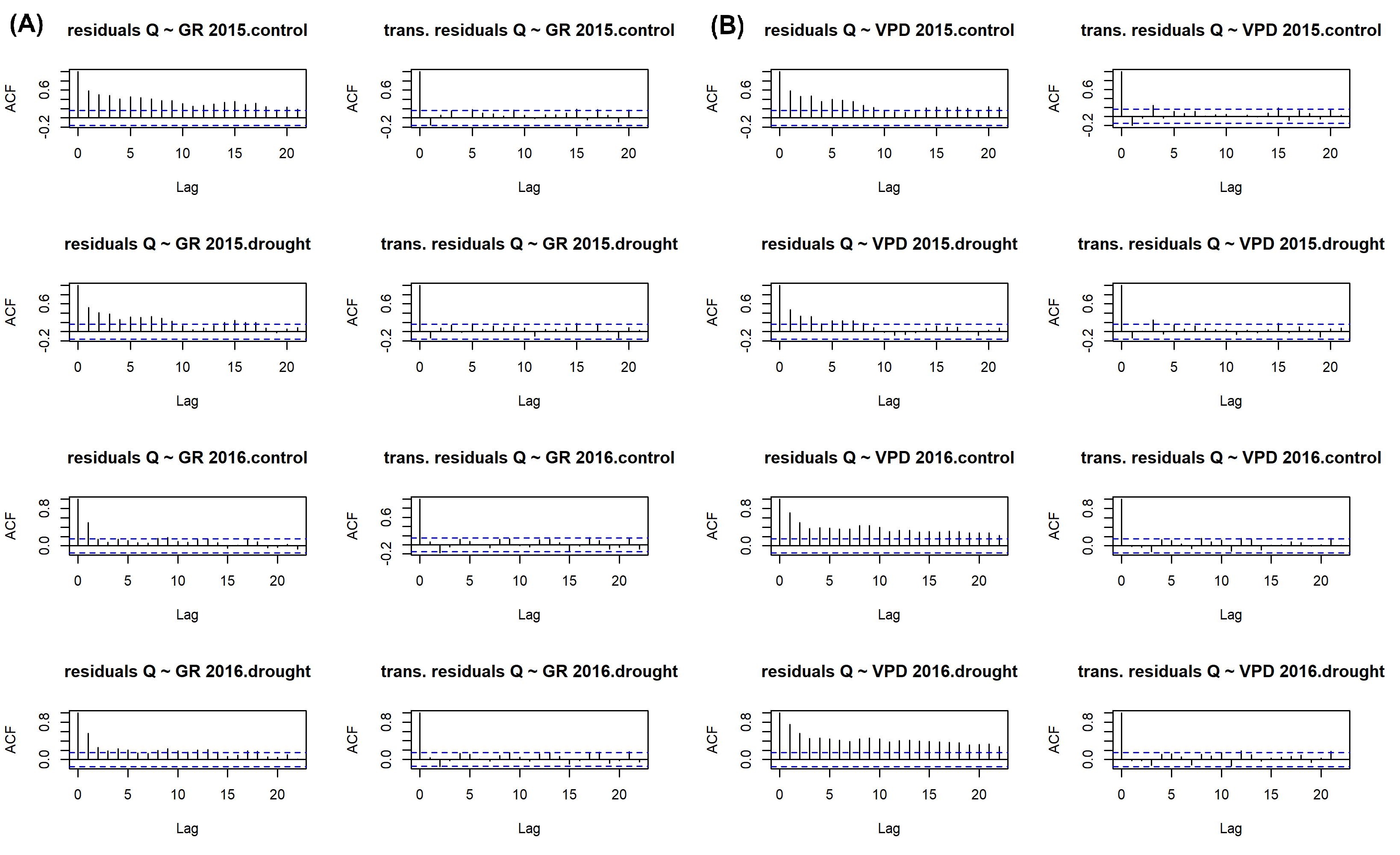

Supplement: Supplementary Figure 5 — Autocorrelation of the models’ residua from the linear relationship of Q with GR (A) and Q with VPD (B) before (left) and after (right) Cochrane-Orcutt transformation. [file Image_5.jpeg]

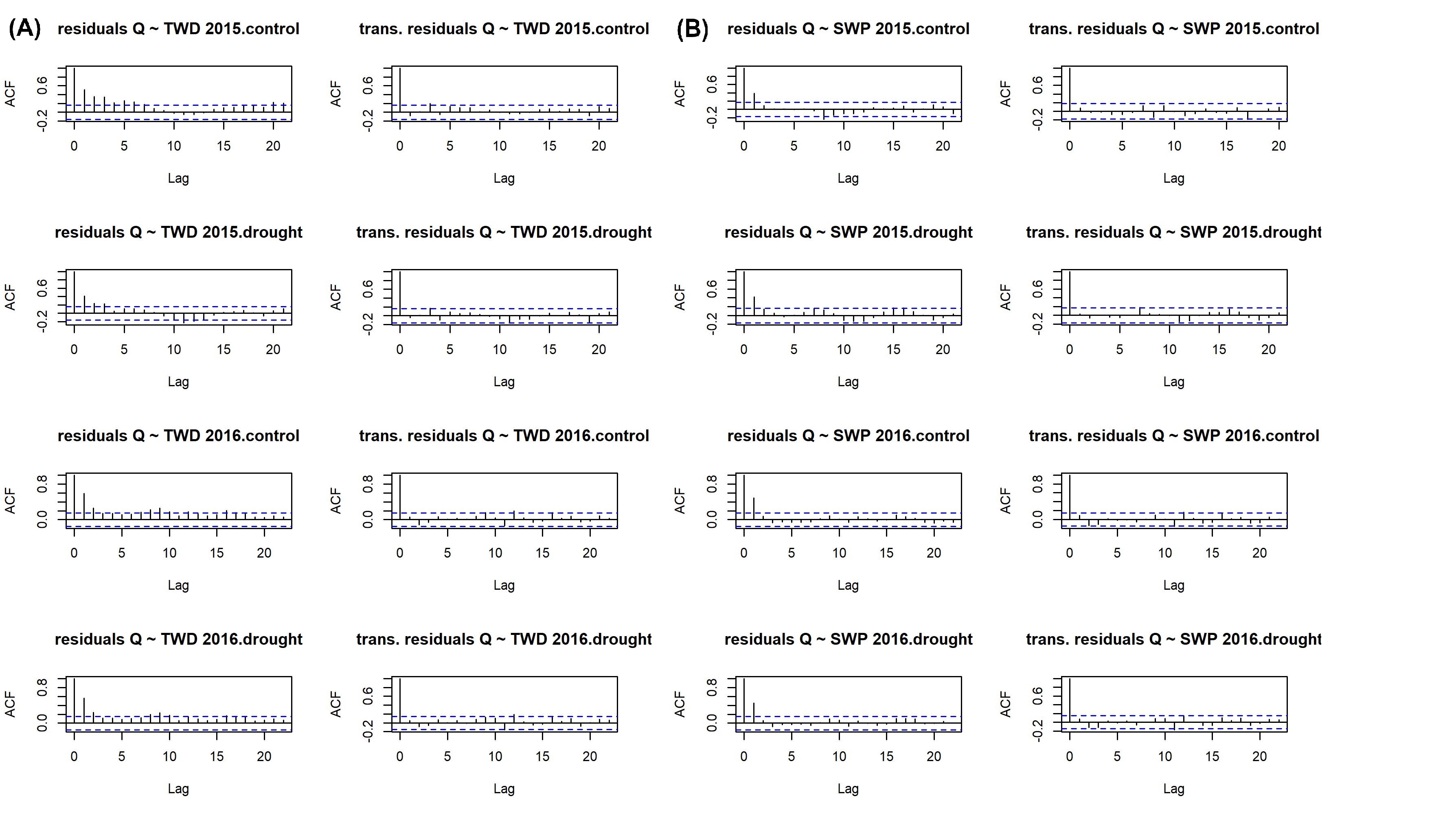

Supplement: Supplementary Figure 6 — Autocorrelation of the models’ residua from the linear relationship of Q with TWD (A) and Q with SWP (B) before (left) and after (right) Cochrane-Orcutt transformation. [file Image_6.jpeg]
